# Is Periodontitis Associated with Age-Related Cognitive Impairment? The Systematic Review, Confounders Assessment and Meta-Analysis of Clinical Studies

**DOI:** 10.3390/ijms232315320

**Published:** 2022-12-05

**Authors:** Arkadiusz Dziedzic

**Affiliations:** Department of Conservative Dentistry with Endodontics, Medical University of Silesia, 40-055 Katowice, Poland; adziedzic@sum.edu.pl

**Keywords:** periodontitis, cognitive impairment, cognitive decline, dementia, Alzheimer’s disease, systematic review, bias analysis, meta-analysis, systemic impact, oral inflammatory disease

## Abstract

It has been suggested that molecular pathological mechanisms responsible for periodontitis can be linked with biochemical alterations in neurodegenerative disorders. Hypothetically, chronic systemic inflammation as a response to periodontitis plays a role in the etiology of cognitive impairment. This study aimed to determine whether periodontitis (PDS) is a risk factor for age-related cognitive impairment (ACI) based on evidence of clinical studies. A comprehensive, structured systematic review of existing data adhering to the Preferred Reporting Items for Systematic Review and Meta Analyses (PRISMA) guidelines was carried out. Five electronic databases, PubMed, Embase, Scopus, Web of Science, and Cochrane, were searched for key terms published in peer-reviewed journals until January 2021. The Newcastle–Ottawa scale was used to assess the quality of studies and risk of bias. The primary and residual confounders were explored and evaluated. A meta-analysis synthesizing quantitative data was carried out using a random-effects model. Seventeen clinical studies were identified, including 14 cohort, one cross-sectional, and two case-control studies. Study samples ranged from 85 to 262,349 subjects, with follow-up between 2 and 32 years, and age above 45 years, except for two studies. The findings of studies suggesting the PDS-ACI relationship revealed substantial differences in design and methods. A noticeable variation related to the treatment of confounders was observed. Quality assessment unveiled a moderate quality of evidence and risk of bias. The subgroups meta-analysis and pooled sensitivity analysis of results from seven eligible studies demonstrated overall that the presence of PDS is associated with an increased risk of incidence of cognitive impairment (OR = 1.36, 95% CI 1.03–1.79), particularly dementia (OR = 1.39, 95% CI 1.02–1.88) and Alzheimer’s disease (OR = 1.03 95% CI 0.98–1.07)). However, a considerable heterogeneity of synthesized data (I^2^ = 96%) and potential publication bias might affect obtained results. While there is a moderate statistical association between periodontitis and dementia, as well as Alzheimer’s disease, the risk of bias in the evidence prevents conclusions being drawn about the role of periodontitis as a risk factor for age-related cognitive impairment.

## 1. Introduction

Aging is often associated with a higher prevalence of chronic inflammatory diseases and co-morbidities, including neurogenerative diseases, especially ACI. The age-related changes contribute to the development of chronic diseases [1,2]. More than 50 million people (5.2% of the global population) face senility with disabling neurodegenerative disorders, such as dementia, commonly associated with Alzheimer’s disease (World Health Organization, WHO, 2017, [3]). According to WHO and the Alzheimer’s Association (UK), 15–25% of people over 65 years develop mild cognitive impairment at some point in their life, and this trend is increasing worldwide. Over the last decades, a potential association between periodontitis and ACI has been reported and claimed based on clinical and in vitro studies results. It has been hypothesized that a low-grade chronic periodontal inflammation has an impact on the central nervous system (CNS) via several pathological interactions [4,5], including systemic inflammation affecting the CNS and direct invasion of periodontal pathogens or their products [6]. There is therefore a need for critical data analysis of the presence and magnitude of association between these two conditions. The hypothetical relationship between the status of periodontium and cognitive state has been thoroughly investigated over the last two decades using systematic reviews [7,8,9,10].

Reportedly, the clinical and animal-model evidence suggests an association between PDS and ACI based on inflammatory-microbial theory and shared risk factors. The microorganisms involved in periodontitis onset and progression can play a role in pathological mechanisms of chronic neurodegenerative conditions, such as ACI. As a result of an extended lifespan and demographic change, the prevalence of age-related chronic diseases has been increasing [11,12]. It is predicted that by 2050, older adults will comprise one-fifth of the world population [13]. Ageing is a non-modifiable risk factor for many chronic illnesses [14,15], resulting in a rise in healthcare expenditure and global healthcare burden [16,17,18]. The chronic peripheral inflammation that occurs in periodontitis and accumulates over the lifespan may contribute to onset and development of systemic diseases. It has been hypothesized that the microbiome of dental plaque and PDS-related pathogens may perpetuate a range of systemic inflammatory conditions, including ACI [4,6]. The role of periodontitis as a risk factor for ACI, manifested by dementia and Alzheimer’s disease, is under investigation.

Periodontal diseases and their most advanced form, periodontitis, are the most common oral diseases across the world [3,19], and reflect the global burden of chronic health conditions [20]. Considering the prevalence of PDS, it is estimated to affect about 20–50% of the global population [19,21]. Centers for Disease and Control (CDC) data reported high prevalence of PDS in the United States in almost half of the nation (47.2%) of adults above 30 years and older have experienced some form of PDS, which increases with age, and 70.1% of adults 65 years and older have periodontal disease [22]. As PDS is a multifactorial inflammatory condition caused by the pathogenic biofilm, dysbiotic oral microbiome, and autoimmune dysregulation [23,24,25,26], the host response is a complex interplay between numerous cells and inflammatory mediators. A hyper-inflammatory response to antigens, lipopolysaccharides (LPS), and bacterial exotoxins, e.g., gingipain produced by the *Porphyromonas gingivalis* pathogen causes ‘hyper’-production’ of systemic mediators (OPT, RANKL, ICAM-1), cytokines (TNF-α, INF-γ, IL-1, IL-6, PGE2), prostanoids, and metalloproteinases (MMPS). Subsequent oxidative stress leads to chronic periodontal inflammation damaging connective tissues [25,27,28]. The regulation leading to the release of proinflammatory cytokines from a variety of cells, such as macrophages, PMN leucocytes, and lymphocytes, is activated by pathogen-associated molecular patterns such as LPS [29,30].

### 1.1. The Bidirectional, Circumstantial Relationship, Shared Risk Factors for Periodontitis and Cognitive Impairment

**Figure 1:**
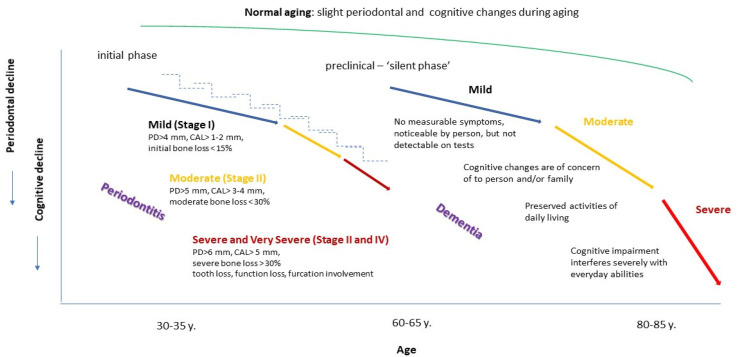
The timeline continuum of periodontitis versus age-related cognitive impairment. The aging process unequally confounds the time available for periodontitis and age-related cognitive impairment initiation over a lifespan.

The complex interrelationship between PDS and ACI seems bidirectional as persons with ACI have inadequate oral hygiene, adversely affecting oral health, including periodontal diseases [31]. The current evidence is deemed to be supporting the hypothesis that ACI is a risk factor for PDS [32,33,34,35], however, the quality and strength of this evidence remains questionable. The link between ACI, tooth loss, and periodontitis has been demonstrated in numerous systematic reviews [36,37,38,39]. Conversely, deteriorating oral health, including tooth loss, periodontal disease, and edentulousness, was associated with a higher risk of developing cognitive decline [40]. A cohort study observed that periodontitis was related to a six-fold increase of cognitive impairment rate and an increase of serum pro-inflammatory markers in persons with Alzheimer’s disease [41]. The authors hypothesize that the active stage of PDS is essential in exacerbating cognitive impairment once it is established. These findings must be interpreted with caution, as the follow-ups duration do not meet criteria for longitudinal cohort studies. Compared to the epidemiology of ACI, due to the earlier onset, the higher prevalence of periodontitis is observed [42] (Figure 1). According to Global Burden Disease database, the global temporal trend of PDS seems to have remained relatively constant for population 50+ years old over the recent decade in the period 2004–2012. However, the prevalence of dementia is expected to accelerate and increase by 2050 to 100 million [43]. The prevalence of PDS and ACI increases with age [44,45]. The onset of generalized PDS occurs between 22–28 years of age on average [46], while the average age at the onset of dementia is estimated to be 80 years, with early-onset dementia occurring before 60–65 (Figure 1).

**Figure 2:**
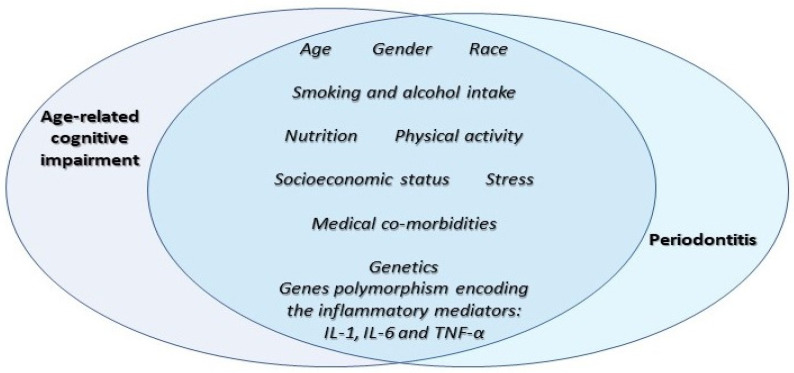
Shared risk factors and predictors for periodontitis and age-related cognitive impairment. Lifestyle, environmental determinants, and genetic predisposition.

Early epidemiological association studies [47,48] observed that elderly persons with cognitive decline were found to have a higher prevalence of oral health problems, including periodontal disease. Subsequent studies have highlighted a bilateral correlation, as periodontitis can be associated with ACI, and tried to look for biologically plausible mechanisms. As both PDS and ACI share risk factors (determinants), possible confounding factors could explain hypothetical association, including age, smoking, diet, and other risk factors. Cardiovascular episodes and strokes can be linked to periodontitis leading to direct effect on a cognitive function [49]. Initially, the advanced destruction of periodontal structures observed in older age was deemed as a consequence of cumulative effect over a lifespan, not a contributor or a result of specifically increased rates of destruction [50,51,52]. Unlike ACI, the main causative factors of periodontitis are microorganisms [53], however both conditions share lifestyle and environmental etiological factors, including the immune response and genetics [54,55] (Figure 2).

It has been hypothesized that a low-grade oral chronic infection impacts systemic functioning. These interactions can be associated with various biological mechanisms, complex signaling systems, and bloodstream transmission [56,57]. Although the blood–brain barrier protects the CNS against microorganisms and inflammatory mediators [58], there is a dynamic change of immune compensation and physiological integrity [59]. Immunologic, biochemical, and microbiologic studies suggest a causal role for periodontitis on ACI, whereby periodontal pathogens (periopathogens) are capable of triggering inflammation and affecting neural structures [33,60,61]. The non-specific inflammatory reactions in CNS are deemed to play a role in ACI pathophysiology [62]. The inflammatory-microbial theories [33,63,64] are currently under investigation. Two main pathological interactions have been proposed to explain interconnections between PDS and ACI [61,65,66]: (1) Systemic inflammation affects the central nervous system due to chronic peripheral periodontal inflammation, host response, and increased pro-inflammatory cytokine levels, (2) Direct invasion of periodontal pathogens and their products into the cerebral region via the circulatory system, triggering a cascade of inflammatory responses within brain tissue.

### 1.2. Induction of Systemic Immune Response Because of Periodontal Inflammation

As periodontitis involves inflammatory mechanisms and immune response of the host, it could have systemic implications [67,68,69]. Chronic oral inflammation (PDS) may lead to systemic overactivity, contributing to neurodegeneration [61,70]. Similar biological pathways have been used to link PDS to cardiovascular disease [49,71]. Several hypothetical associations between PDS and systemic status have been proposed [44,56,72], including oxidative stress, endothelial malfunction and/or systemic inflammatory response as a result of periopathogens bacteraemia and mediators release from periodontal tissue [73,74]. Reportedly, studies suggested that the existing PDS with associated serum IgG antibodies to main periopathogen *Porphyromonas gingivalis* were linked to elevated proinflammatory cytokine level, C-reactive protein, and TNF in individuals with cognitive decline [60,75,76,77]. The exact underlying mechanisms have not been verified.

A non-specific inflammation within the neural structures secondary to other pathological processes may lead to cerebral neurodegeneration [78,79]. Attempts have been made to demonstrate the inter-relationships between unresolved inflammatory condition and the immune response shifts in immune-privileged tissues inducing chronic illnesses [80,81]. Periodontitis is a substantial source of antigens interacting with immune defense mechanisms and triggering inflammatory reactions [77,82], including the sustained release of endogenic inflammatory mediators, such as cytokines, interleukins, and their increased level in circulation [83,84,85,86]. A hyper-responsive phenotype is responsible for the susceptibility to some chronic diseases, comprising asthma, chronic obstructive pulmonary disease, lupus erythematous, rheumatoid arthritis, as well as a chronic periodontal infection. This individual predisposition appears to be the causative factor of aggravated inflammatory reactions to periopathogens. Similarly, a phenotype-based association might be linked to ACI [87]. Systemic inflammation has been indicated as a risk factor for Alzheimer’s disease [80,88,89]. A ‘vicious circle’ of inflammatory pathomechanisms, including autoimmune responses and the continuous suppression of compensating mechanisms, could explain the deterioration of both cognitive functions and periodontal health [60,61].

**Figure 3:**
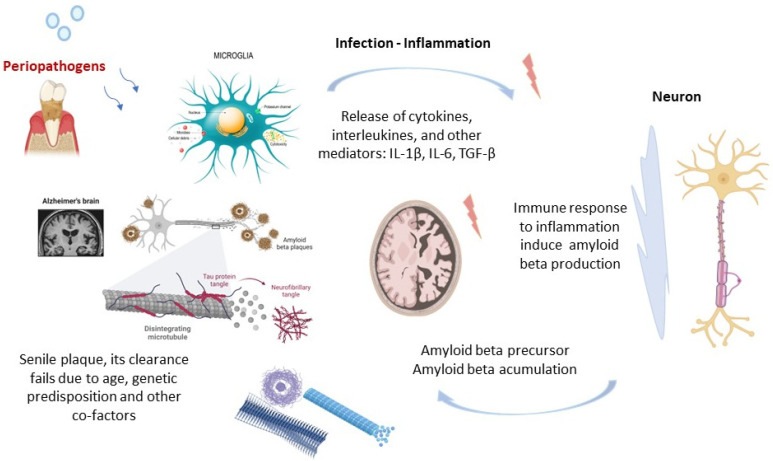
Hypothetical mechanism of the neural pathological changes associated with chronic periodontal inflammation [65], Biorender platform. The potential suggested primary role of periodontal pathogens, inflammatory mediators, local and systemic immune response, with a subsequent induction of amyloid beta formation and senile plaque accumulation in the central nervous system.

A periodontitis-induced immune response may cause the aggregation of beta-amyloid (Aβ) and hyperphosphorylated tau proteins accumulating in senile cerebral plaques and create neurofibrillary tangles [90]. Aβ may be an antimicrobial molecule that initiates immune reactivity, a first-line protective factor against pathogens [91]. The cytokine release and hyper-inflammatory response of dysregulated immune defense mechanisms in PDS have been reported to aggravate autoimmune systemic reactions in various parts of the body, including brain tissue [61,92,93]. This includes pro-inflammatory cytokines (interleukin 1, interleukin 6, tumor necrosis factor alpha, and acute-phase proteins) that control intercellular signaling [79,94] that have been attributed to PDS [86] and noted in neurodegenerative diseases [95,96,97]. Potentially, a transient neural infection and subsequent cerebral inflammation might be a trigger of a prolonged self-perpetuating innate immune response, resulting in gradual neurodegeneration [61,98] (Figure 3). The genes linked to ACI are believed to cause Aβ retention without a trigger from the innate immune components. Individuals with the apolipoprotein E4 (APOE-ε4) gene associated with slow-onset Alzheimer’s disease may be more susceptible to ACI [99]. A possible role of periopathogens in the pathology of Alzheimer’s disease is yet to be explored [64].

It was speculated that some neurodegenerative conditions may be associated with microbial vectors [65,100,101] and direct local immune overstimulation (Supplementary Table S1). Periodontal pathogens could initiate or exacerbate cerebral inflammation, contributing to ACI onset and/or progression [102,103]. Whilst specific infectious vectors have not been established, the innate immune system can be stimulated by infection of the neural tissue with periodontal microorganisms to aggravate the beta-amyloid cascade (Figure 3) [104,105,106].

Current data concerning the pathogenesis of neurodegenerative diseases, such as Alzheimer’s disease [101,107] and Parkinson’s disease [108,109], suggest that microorganisms may be responsible for immune overreaction. As plaque accumulation and gingival inflammation allow periopathogens to enter the systemic circulation [110], it may increase the risk of developing medical conditions [111,112]. These mechanisms involve the increased vascular permeability and damage of the oral epithelium caused by periopathogens [113]. A dysregulated immune system and the contributing role of impaired barriers play an important role in pathogenesis of ACI [114,115]. Importantly, the biomolecule lipopolysaccharide (LPS) of *Porphyromonas gingivalis* (*P. gingivalis*) is capable of crossing the blood–brain barrier in ACI brain samples [70,116], whereas cognitive impairment induced by *P. gingivalis* was claimed to be associated with neuroinflammation [117,118]. This finding was replicated in animal studies, which showed that the demyelinating encephalitis CNS condition deteriorated following the inoculation of *P. gingivalis* [93]. Persons with a cognitive decline had increased levels of antibodies against *P. gingivalis* as an inflammatory systemic response [34,36]. Besides, as periopathogens reveal certain neurotropic abilities, they may affect neural function [6,31]. Arguably, selected oral microorganisms (spirochetes) can invade CNS via peripheral nerves and trigeminal ganglion [119]. Different periodontal pathogens have been found in post-mortem cerebral specimens in ACI, with frequent isolation of periodontal spirochetes [63,66,70,101,120]. Particularly, anaerobic *Treponema spirochetes*, specifically *T. denticola*, *T. pectinovorum*, *T. vincenti*, *T. amylovorum*, *T. maltophilum*, and *T. socranskii*, were detected in the brain tissue of patients with Alzheimer’s disease. These pathogens can induce amyloid plaque formation in an animal model [120,121] (Supplement Materials, Table S1).

### 1.3. Rationale for the Systematic Review (SR) Project

ACI is an incurable neurological condition, which in its advanced stage affects quality of life and life longevity. Hence, considering the potential influence of periodontitis on systemic conditions, including neuroinflammation, a detailed appraisal of existing evidence of potential ACI risk factors is justified, while inconsistent scientific information has been produced concerning the role of periodontitis in ACI.

The aim of this comprehensive research project was to determine whether periodontitis is a risk factor for age-related cognitive impairment. This critical review with a systematic approach aims had three objectives: (1) to identify and appraise the quality of existing scientific evidence, (2) to determine the potential impact of common confounders and shared risk factors of periodontitis and age-related cognitive impairment on the relationship between the two conditions, and (3) to evaluate the direction and magnitude of any relationship between periodontitis and age-related cognitive impairment. It is expected that the obtained results provide an impetus for further studies which can deliver conclusive data related to ACI causation.

## 2. Materials and Methods

A comprehensive review utilizing a systematic approach was conducted in adherence to the Preferred Reporting Items for Systematic Review and Meta Analyses (PRISMA) guidelines [122,123]. A primary investigator (A.D.) screened all records, with a support from library, University of Bristol, and supervision (P.R.), assessing data extraction, quality assessment, risk of bias, and completing meta-analysis as integral parts of individual MSc research project. For observational studies, a simplified Population, Intervention, Comparator, and Outcome (PICO) protocol [124,125] was formulated as follows: (A) Population: adults who are 45 years or older, (B) Intervention (exposure): periodontitis defined by clinical and radiological parameters such as: probing pocket depth, clinical attachment loss, or alveolar bone loss, (C) Comparator: adults, 45 years or older, without clinically diagnosed periodontitis, (D) Outcome: cognitive impairment, cognitive decline, including dementia as a result of Alzheimer’s disease, where the primary cognitive defect was not a result of a congenital/genetic condition or acquired cerebrovascular episodes affecting CNS function.

### 2.1. Database Search Strategy

PubMed/Medline, EMBASE, Scopus, Web of Science, and Cochrane electronic databases were searched without year restriction since the classification system of periodontal disease containing modern diagnostic protocols was introduced. The additional Google Scholar and Clinical Trials online sources were also reviewed via study topics. The relevant and appropriate keywords were applied, including MESH terms. The primary search was intended to identify any articles published and available online until 2021 that involved clinical studies on periodontitis and cognitive impairment, with particular attention on Alzheimer’s disease and dementia (Table S2).

### 2.2. Studies Selection

The search was conducted by A.D., with the support of the University of Bristol library staff and research project’s supervisor (P.R.). The databases were searched, and the results obtained were screened in two steps. After initially screening the titles, the abstracts were reviewed to find only those that met the inclusion criteria. Next, the full content of these selected articles was downloaded, with particular focus placed on the clinical studies and methodology. To maximize the search, the reference lists of the selected articles were reviewed for additional sources, collecting a relevant PDS/ACI-related literature for the subsequent structured analysis and data extraction.

### 2.3. Inclusion and Exclusion Criteria

The inclusion criteria included (a) English-language clinical studies published as peer-reviewed articles between 1970 and January 2021, (b) original clinical studies, namely: cross-sectional, case-control, clinical cohort studies, including clinical trials, (c) studies with human participants assessed for periodontitis using clinical intraoral examination and/or radiographs, which included: periodontal/probing pocket depth (PPD), clinical attachment loss (AL), community periodontal index (CPI), and alveolar bone loss (ABL), (d) studies with participants assessed for ACI, cognitive decline, dementia, or Alzheimer’s disease, assessed by standardized, recognized, and verified cognitive function tests, (e) no restrictions regarding gender, ethnicity, socio-economic status, co-existing co-morbidities, or education, (f) study’s participants aged above 45 years old (exemptions if justified for systematic review purpose). The exclusion criteria contained (a) experimental laboratory studies, commentaries, letters, cases, conference presentations, non-scientific articles, theses, and editorials, (b) in vitro studies, studies on animals, and articles that did not include the result of interest, (c) studies in which a diagnosis of periodontitis/periodontal disease and cognitive impairment was established solely based on questionnaires, (d) studies in which periodontitis was not clearly defined or the diagnosis was established on visual criteria or tooth-loss, (e) studies that described clinical intervention in the form of complex periodontal surgery, (f) non-peer-reviewed reports (e.g., pre-prints, preliminary research reports, repositories), (g) studies that included primarily the reverse causal effect and relationship, i.e., the influence of cognitive impairment on oral health, the prevalence of tooth loss alone if it is not possible to extract valid data, (h) studies with periodontitis as a manifestation of congenital/genetic/immune conditions, (i) studies that include participants with a diagnosed cognitive impairment because of congenital/genetic condition, trauma, or cardiovascular incidents.

Selected studies meeting the defined inclusion criteria were included for further data extraction, quality assessment, assessment risk of bias, and confounders evaluation. The data gathered from each study were extracted using Microsoft Excel and Microsoft Word to create worksheets with the details, and Endnote X.7.1 v was used to manage the processing of references (Table S3). Data extraction allowed obtaining the necessary information about study characteristics and findings from included observational studies.

### 2.4. Quality Assessment of Studies Risk of Bias Evaluation

The Newcastle–Ottawa Scale (NOS) was applied to assess the quality of observational studies (certainty of evidence), and indirectly a risk of bias [126]. The NOS scores from 1 (minimum) to 9 (maximum) in three domains: (1) study participant selection and design; (2) comparability of participant groups; and (3) assessment of outcome (exposure). Articles that scored 9 or 10 were classified as ‘high quality’, those valued at 7 or 8 were deemed ‘moderate quality’, and those with value 6 or lower were ‘low quality’.

### 2.5. Assessment of Confounding

Baseline characteristics of the studies included the comparison of the main confounders in each study. This approach allowed the identification of whether authors used any methods to control selection bias. Qualitative assessment of these confounders and potential residual confounders occurring in each reviewed article verified (1) the presence/absence of methods for confounders control (stratification/standardization), (2) demonstration of the sufficient balance between groups for the confounder, (3) adjustment for the confounding using statistical modelling, e.g., stratified analysis, multiple logistic regression, multivariable regression analysis, and Cox proportional hazards regression. 

### 2.6. Data synthesis and Statistical Analysis

Multiple meta-analyses of extracted quantitative data using a random-effects model were conducted for studies that included suitable statistics, supported by RevMan v5.4.1 software (Cochrane Collaboration’s software, Oxford, UK) [127]. The association between PDS and ACI was statistically estimated based on the odds ratio (OR) values at 95% confidence interval (CI), enabling quantification of cumulative adjusted and non-adjusted risk of ACI in correlation with PDS. Heterogeneity characteristics among studies have been quantified by applying I2 statistical analysis. The I2 value above 75% and *p* value > 0.05 showed a high heterogeneity, when values 25% and 50% were considered as low and medium heterogeneity [128,129]. The measured weights accounted for the variations in study participants and designs, indicating the population’s inferences. Pooled data were calculated for all types of cognitive impairment/dementia, regardless of its severity, and all forms of periodontitis (mild, moderate, severe).

## 3. Results

**Figure 4:**
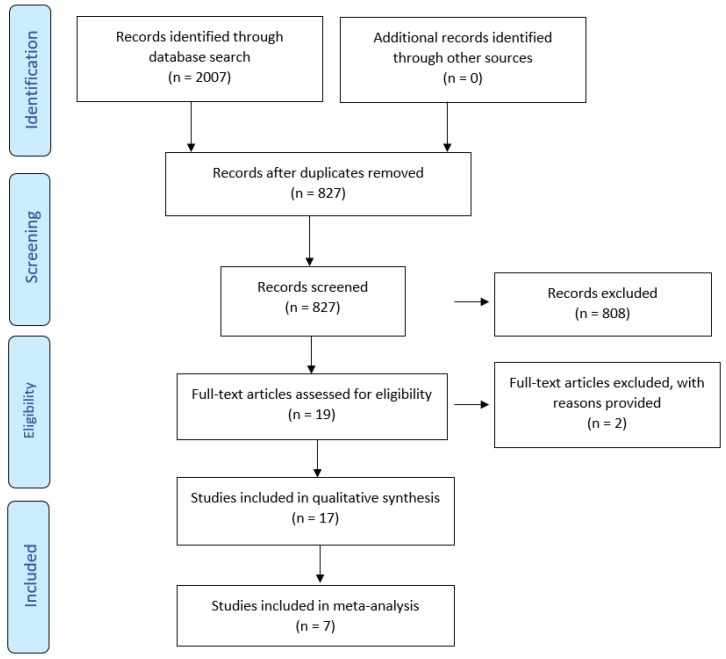
PRISMA flow diagram for systematic review summarizing the results of searches of electronic databases [122,123]. Identification of studies via databases using keywords and MESH terms. Total number across all databases, records included as per eligibility criteria, records excluded, articles included in meta-analysis (data synthesis).

The comprehensive search using electronic databases identified 827 potential sources. After screening abstracts and removing duplications and exclusions, 19 sources remained that met the inclusion criteria (PRISMA protocol, Figure 4) [122,123]. Two potentially relevant studies [41,130] were excluded due to the unclear periodontitis diagnostic criteria, not matching the outcome of identified studies. Additionally, no clear follow-up, potential issues with excluded exposure, and the likelihood of impaired cognition at baseline were noted.

### 3.1. Characteristics of Included Studies

Of 17 studies identified, 14 were cohort studies, one was cross-sectional, and two were case control. The design declared by the authors was revised, based on their protocol and methods. All those declared as ‘retrospective’ were reclassified as prospective. One declared as ‘case-control’ was classified as ‘cross-sectional’ [131]. Table 1 summarizes the studies in terms of design and sample size, duration, and outcomes. The cohort studies were carried out in Taiwan [132,133,134,135,136], South Korea [137], Japan [138,139,140], the USA [141,142,143], France [144], and Sweden [145]. Sample sizes ranged from 85 [139] to 262,349 [137], with follow-up between 3 [139] and 32 years [143] (mean: 10 years). Twelve studies included both genders, and one study concerned only males—military veterans [143]. Except for two studies ([143], age 28–84; and [136], age > 20 years), all others included middle-aged and elderly individuals, aged over 45 years.

One cross-sectional study, including 154 participants of each gender, was performed in the USA [131], with a study period 3 years, and the subjects’ age > 45 years. The two case-control studies were conducted in the USA [146] and Spain [147], with 144 and 409 participants, respectively, and widely different durations. The case group-specific study performed by Stein et al. [146] was limited to females only (nuns). The follow-ups intervals for periodontal status and cognitive function assessments in studies with a longer duration were highly heterogeneous, from annual evaluations to a baseline until final point assessment only.

### 3.2. Comparison of Outcome Measures and Diagnostic Criteria

Some outcomes in the cohort studies were the neurological diagnosis of new-onset dementia, mild cognitive impairment, cognitive function decline, development of mild memory impairment, dementia, and Alzheimer’s disease incidence as various primary outcomes, in correlation with periodontal status (Table 2). Cognitive function decline/progression was used in one study [139]. Four studies also included incidence of vascular dementia [135,136,137,144]. Fourteen studies focused on dementia or cognitive decline incidence, whereas only two cohorts [136,140], and one cross-sectional study [130] considered Alzheimer’s disease incidence as the outcome. Five cohort studies in Asian countries used similar methods of data collection, extracted from the national health care system [131,132,133,134,135,136]. One study [135] assessed the risk of developing dementia in individuals undergoing periodontal treatment.

A wide selection of diagnostic criteria were applied as primary and secondary outcome measures to diagnose the outcomes (Table 3). The risk of developing dementia and cognitive decline were predominantly investigated as primary measures, whereas the occurrence of Alzheimer’s disease was assessed in two cohort studies [133,137]. Table 3 presents the diagnostic criteria for ACI used in 17 clinical studies. The diagnostic criteria for PDS also varied appreciably (Table 3), involving clinical and/or radiological parameters. Four conceptually similar studies used standardized periodontal clinical indices: probing pocket depth (PPD) and clinical attachment loss (AL) exclusively, however not sufficient if used alone, while other four studies optimally assessed PPD and additionally alveolar bone loss (ABL) as the recommended diagnostic standard [131,143,145,147]. The simplified Community Periodontal Index (CPI) and its integral component PPD (codes 3 and 4 cut off) were also used in two prospective studies [134,138].

Most studies included mainly combinations of periodontium assessments, such as PPD, AL, ABL, bleeding on probing (BOP), and periodontal inflamed surface area (Table 3). A comprehensive radiological examination of ABL (3-stage) using orthopantomograms was used in three studies [131,146,147]. Six studies primarily used the WHO ICD-9-CM basic epidemiological classification (Table 3). The definition of periodontitis according to European Workshop in Periodontology Group C (EWP) and Centers for Disease Control/American Academy of Periodontology (CDC/AAP) was also applied [138,139]. Periodontal assessment was performed mostly by ‘calibrated’, trained dentists, whereas cognitive state examination was executed by neurologists, psychiatrists, and/or clinical psychologists.

### 3.3. Quality Assessment of Reviewed, Observational Studies

The quality of evidence (certainty) and risk of bias using the Newcastle–Ottawa scale (NOS) are presented in Table 4, Table 5 and Table 6 [126]. The overall quality of studies of cohort, longitudinal studies were moderate, with a mean score 5.8 (range 5–7). In cross-sectional and case-control studies the quality assessment results were similar (moderate score), with a mean score of 5.25, a minimum value of 5, and a maximum value of 6. None of the selected studies achieved the highest scores of 8 or 9. The lowest quality related to non-standardized methods’ protocol was observed in case-control studies [131,146]. A substantial risk of bias has been observed in some cohort studies, with the primary concerns being lack of representativeness of exposed cohort (nine studies). Similarly, quality assessment showed a risk of bias in one cross-sectional and one case-control study regarding representativeness of the sample. The studies were of variable quality and methods were not clearly presented to reach conclusions regarding certain quality criteria. The comparability criterion was suboptimal in all studies, although they were controlled for primary confounders such as age and gender. Other important confounders and residual ones (smoking/alcohol, socio-economic status) were omitted or analyzed insufficiently. A detailed consideration of the treatment of confounders is presented below (Table 7 and Table S4). The least satisfactory criterion was representativeness. However, in a controlled study, comparability has a more impactful effect.

### 3.4. Adjustment for Confounding, Measurement of Confounders

Confounders taken into consideration by the source studies are summarized in Table S2. The main cofounders and covariates considered in the source studies were age, gender, smoking, alcohol intake, comorbidities (mainly cerebrovascular diseases, hypertension, cardiovascular diseases, and diabetes), and education level (various criteria). The analysis was adjusted for co-existing comorbidities in 15 studies. Studies varied widely on the number of cofounding factors and covariates considered, from three [132,134] to eleven [131]. The least common ones included physical activity, income, oral habits, and oral hygiene/dental care [131,147]. Interestingly, five cohort studies did not consider smoking or alcohol intake as confounders [132,134,135,136]. Various methods of controlling confounders were applied during study design and analysis (Table 7). Age and gender were matched between groups in some studies [132,133,134]. Multivariate regression models were mainly used to reduce the influence of confounders (Table 7). These allowed for the extraction of adjusted odds ratio (OR) and risk ratio (RR).

The treatment of residual confounding varied considerably between studies (Table 8), and a wide range of criteria were applied for socio-economic status. Education level was ignored or dichotomized [138,140], but not analyzed as a numeric or ordinal variable. Most studies used a similar categorization scale for smoking, i.e., ‘never’, ‘former/previous’, and ‘current’. There was a great scope for residual confounding, as some categories were inaccurately defined (Table 8). In the longitudinal study carried out by Iwasaki et al. [139], the education covariate did not affect a level of association between primary variables based on stratification method due to remaining confounding within each educational stratum. In general, the treatment of selected essential confounders appeared largely inadequate in quantify studies, with the additional risk of not sufficiently treated residual confounders.

### 3.5. Summary of Systamatic Review Results Subgroups Analyses

The main results of all 17 studies are compared in Table 1 and Table 2. Overall, positive results were noted in 13 studies (11 prospective, one cross-sectional, and one case-control study), while no significant association was found in three cohort studies [137,138,141] and one case-control study [146]. These four studies, with durations between 5 and 15 years, encompassing 144 to 2335 cases, reported that persons with periodontitis did not demonstrate an increased risk of ACI. The results of one study partially supported a positive association for selected periodontal parameters [142]. After study duration adjustment and inclusion of studies with at least 10 years follow-up period, in 7 reviewed studies a positive correlation was found, with the total number of cases ranging from 597 [143] to 262,349 [137], and with study duration between 10 and 32 years (mean: 14.3 years). Direct comparison of quantitative data from all included studies was unachievable due to the range of measures of effect (hazard ratios, odds ratios, risk ratios, means, percentage). Seven studies indicated a PDS-ACI relationship by providing HR values, five used OR, and one study described quantitative results with only RR (Table 2). The analysis of the results of thirteen studies plausibly supports the hypothesis of association between PDS and ACI, particularly regarding dementia and Alzheimer’s disease, in populations with existing moderate to severe PDS. Six prospective studies with samples ranging between 6,056 and 262,349 found mild to moderate associations between clinical parameters of periodontitis (PPD, AL, ABL) and various level of cognitive function decline assessed by neurological tests, after controlling for main confounders (Table 2).

Overall, 11 cohort studies, one cross-sectional study, and one case-control study reported a mild or moderate association between periodontitis and ACI, based on difference between cohorts exposed and non-exposed to periodontitis. This relationship was confirmed for different primary outcomes and all types of ACI, including dementia, general cognitive impairment, mild cognitive decline, vascular dementia, and Alzheimer’s disease. The more evident association was observed in studies investigating the link between periodontitis and dementia or cognitive impairment generally, compared to studies including Alzheimer’s disease groups as a primary outcome. The lowest estimated hazard ratio as a risk of ACI among all cohort populations was 1.05 [137] and the highest was 2.54 [136], with a much wider range of odds ratios from 1.50 [142] to 15.12 [131]. In the vast majority of studies, a narrow 95% CI range was noted, demonstrating appropriate accuracy of estimation. One short duration (3 years), cross-sectional study carried out in USA on a relatively small cohort of participants (154) [131] presented disproportionally higher OR (15.12), with a wide CI: 5.93–38.58 while assessing the risk of Alzheimer’s disease, mild cognitive impairment, and subjective cognitive impairment. The discrepancy in OR values among cohort, cross-sectional, and case-control studies raised concern reflecting incompletely controlled confounders.

The age factor indicated an inclination towards ACI with co-existing periodontitis. The risk of dementia was significantly higher in patients with periodontitis who were over 80 (HR: 4.30) [132]. The largest cohort study [137] investigating 262,349 persons over the age of 50 demonstrated that periodontitis was associated with an increased risk of dementia, even after adjustment for lifestyle behaviors. The results of two smallest cohort studies conducted in Japan in 2016 (n = 85) [139] and 2019 (n = 179) [138] showed the association between severe and mild cognitive impairment [141] after adjusting for follow-up period and baseline characteristics (adjusted OR: 2.61). Severe periodontitis was associated with the risk of cognitive function decline (adjusted RR 2.2) [139]. Longitudinal study results obtained by Chen et al. [133] in a Taiwanese cohort group of 18,672 persons revealed that patients with ten years of PDS exhibited a higher risk of Alzheimer’s disease (1.707-fold increase). In a study comprising an unexpectedly wide range of subjects’ age [136], people with periodontitis were more likely to develop dementia (HR: 2.085, adjusted HR: 2.54). One of the first comprehensive clinical studies, with the longest study duration (32 years), [143] concluded that a higher rate of periodontal disease progression independently elevated the risk of lower cognitive test scores in USA army veterans. In the most recent longitudinal cohort study, comprising matching groups of 56,018 subjects over the age of 50 and 13 years of follow-up period, Lee et al. [132] reported an increased incidence of dementia in patients diagnosed with PDS, compared to patients without PDS (5.19 versus 3.02 per 1000 person/year), in both males and females. The authors claimed that periodontitis can be an independent risk factor for dementia.

The results from four studies reported no statistically significant difference regarding primary outcome between cohorts exposed and non-exposed to periodontitis (Table 2). PPD and BOP were not predictors of cognitive decline, or associated with poorer cognitive performance in the cohort study performed in the USA [141]. Similarly, Okamoto et al. [140] found no significant association between CPI code 0–4 and mild memory impairment in Japanese community, while Arrive et al. [144] concluded that poor periodontal status was not associated with the increased risk of dementia in French older community dwellers. In one of the earliest studies in a homogeneous group of nuns, Stein et al. [146] observed that moderate and severe periodontal damage evidenced by alveolar bone loss was not associated with incidence of dementia, after adjusting for age, education, and apoE4.

### 3.6. Quantitative Evaluation Meta-Analysis and Sensitivity Analyses

**Figure 5:**
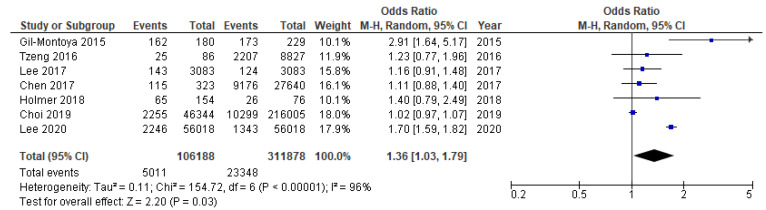
Cumulative meta-analysis results [131,132,133,134,136,137,147]. Forest plot for the association between periodontitis and age-related cognitive impairment in seven eligible clinical studies (Odds Ratio 1.36, 95% CI, 1.03–1.79, high heterogeneity 96%, random effect).

**Figure 6:**
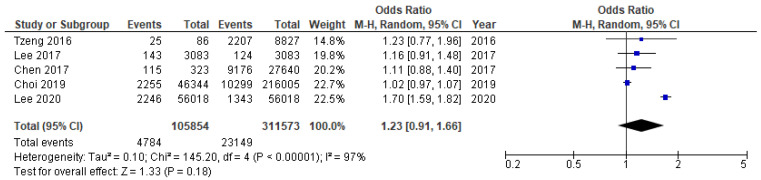
Cumulative meta-analysis results [133,134,135,136,137]. Forest plot for the association between periodontitis and age-related cognitive impairment in five conceptually similar cohort studies (dementia, Alzheimer’s disease, non-specific cognitive decline).

**Figure 7:**
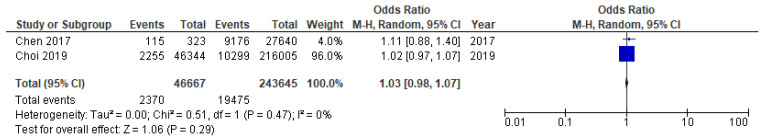
Sensitivity post-hoc analysis to determine the robustness and consistency of main meta-analysis results [133,139]. Forest plot for the association between periodontitis and Alzheimer’s disease (primary outcome) in two cohort studies.

**Figure 8:**
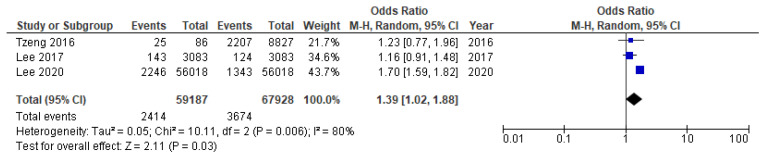
Sensitivity post-hoc analysis to determine the robustness and consistency of main meta-analysis results [134,135,136]. Forest plot for the association between periodontitis and dementia (primary outcome) in three cohort studies.

Meta-analysis synthesized the results of cohort, cross-sectional, and case-control studies. Pooled analysis was carried out separately for studies sufficiently conceptually similar, including the same primary outcome and measures—Alzheimer’s disease (two cohort studies [133,137] and dementia/cognitive decline (three cohort studies [132,134,136]. Finally, five cohort studies [132,133,134,136,137], one cross-sectional [131], and one case control study [147] were combined for meta-analysis. The pooled results for eligible seven studies demonstrated an overall mild association of ACI with periodontitis, with OR = 1.36 (95% CI 1.03–1.79, Figure 5) and RR = 1.20, 95% CI 0.99–1.47. Taking into consideration five cross-sectional studies only, the pooled OR was estimated at lower level—1.23; 95% CI 0.91–1.66, and higher risk difference RD = 1.24 (95% CI 0.91–1.66, Figure 6). The higher OR was calculated for two remaining non-cohort studies (OR = 2.02, 95% CI 0.99–4.14). The OR value for two conceptually similar cohort studies [136,140] regarding independent variable, primary outcome, and measures (Alzheimer’s disease) was estimated at 1.03, 95% CI 0.98–1.07, Figure 7). The OR estimate for the remaining three conceptually similar cohort studies (dementia) was the highest, with 1.39 (95% CI 1.02–1.88, Figure 8). The calculated I2 value 96–97% revealed a substantial heterogeneity between reviewed studies. Hence, the estimation of the overall data from studies included in meta-analysis must be interpreted with caution. All studies were comparable considering the study weight (10.1–18.0%).

**Figure 9:**
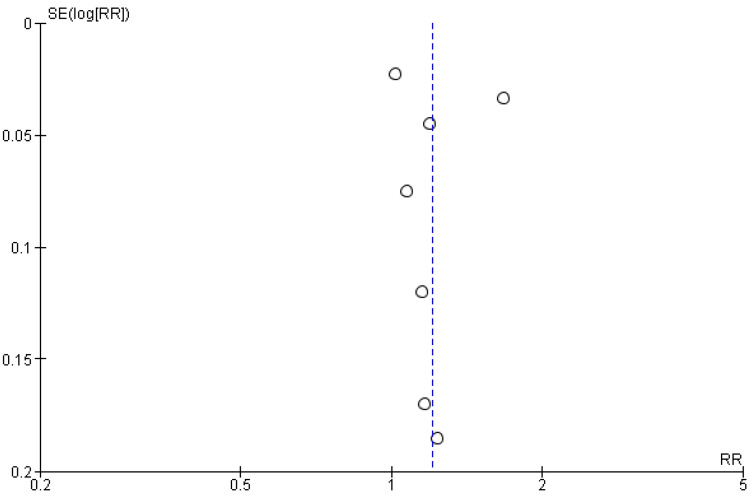
Funnel scatter plot assessing publication bias for seven eligible clinical studies. Potential evidence of publication bias. A graphical representation of the effect estimates from seven studies against measure of each study’s size/precision.

The publication bias and small-study effects were assessed using a funnel plot and Egger’s regression asymmetry test. The funnel plot containing seven eligible studies showed potential evidence of publication bias (Figure 9). This interpretation must be analyzed with caution, while the Begg’s and Egger’s method [148,149] is applied preferably in the presence of more than 10 study results.

## 4. Discussion

This multifaceted systematic review aimed to determine whether periodontitis is a potential risk factor for developing ACI by evaluating the existing evidence. Qualitative and quantitative analysis of literature revealed an apparent moderate association between periodontitis (all stages, mild/moderate/severe) and ACI, including the most common forms of cognitive decline: Alzheimer’s disease, vascular dementia, and mild cognitive impairment. This link was established for dementia and Alzheimer’s disease in 13 clinical studies with different protocol, design, sample size, study duration, and various confounders. However, causality cannot be inferred due to the moderate and, in some cases, low quality of the study designs.

Noteworthy, the obtained study results can be hindered by multiple problems, including: (1) the multi-factorial etiology of PDS and ACI, (2) the wide range of risk factors, (3) different diagnostic criteria for PDS and ACI, (4) the chronic nature of both conditions, which requires longitudinal studies, and (5) ethical aspects related to interventional research projects. SRs of the association between periodontitis and neurological conditions use evidence from observational studies, thus the inferences drawn need to be examined for confounding [150] when other factor(s) other than primary variable of interest of being investigated can directly influence the measured outcome [151]. It may involve a possibility that the observed relationship reflected differences between the study and control groups other than the exposure being investigated. Analyzing the association between periodontitis and systemic health, various determinants, such as smoking, gender, age, and socio-economic status may interfere and confound this association as potential risk factors, independently linked with periodontitis. Due to complicated etiologies of PDS and ACI, clinical studies inevitably lack data on some distorting factors. Confounding can be controlled by appropriate data adjustment such as stratification and statistical modelling [128], and multivariable regression analysis can be used to test an association when controlling for known potential confounders [152].

None of the reviewed studies met the criteria for longitudinal, large sample projects, with sufficient control of cofounders or standardized outcome measures. Insufficient duration of studies and lack of sample size calculations could lead to data misinterpretation. The apparent heterogenicity observed precludes reliable results and conclusions. Adjustment for confounders and residual was achieved in a few studies [132,133,134,135]. The results of observational studies could be affected by various co-factors specific for certain regions, lifestyles, diets, etc. The outcome measures and diagnostic criteria of periodontitis and ACI applied in those studies were inconsistent, which might not precisely reflect the periodontal/cognitive status. This paucity of analyzed data does not allow for conclusion to be drawn regarding periodontitis as a primary risk factor of ACI.

The results of cross-sectional and case-control studies offer weaker evidence of causality than cohort studies. These concerns were reflected in suboptimal scoring of NOS quality assessment (Table 4 and Table 5), particularly considering representativeness and definition of control. While the pooled OR value assessed in meta-analysis was higher than obtained for cohort studies and estimated at 2.02, these results should be analyzed with caution. In comparison, although, Borsa et al. [153] rated five studies in their SR as good or fair quality using National Institutes of Health’s criteria, the inclusion criteria were unclear, considering a definition of ‘periodontal disease’ vs ‘periodontitis’.

The quality of data extracted likely impacts on the results of meta-analysis. They seem consistent with other systematic reviews [154] that reporting pooled RR = 1.38 in the cohort studies and RR = 2.25 in the case control studies, with results of recent meta-analysis carried out by [155] which showed a strong relationship between PDS and cognitive impairment, with odds ratio 1.77 (95% CI, 1.31–2.38). The most recent systematic review conducted by Borsa et al. [153] showed that periodontitis at baseline was associated with a six-fold raise of cognitive impairment rate, however the follow-up period was inadequate (six months). On the other hand, a weak association between periodontitis and dementia was demonstrated in the meta-analysis conducted by Kapellas et al. [34], with OR estimate 1.17 (*p* = 0.33). The present meta-analysis and data synthesis appears based on the same flawed data. Moreover, in the most recent multi-center, international Mandelian randomization study [154], authors concluded that no convincing evidence was found to underpin the hypothesis that periodontitis is a causal risk factor for Alzheimer’s disease. Brignardello-Petersen [155] critically apprised a cohort study conducted by Iwasaki et al. [138] and stated that it has a high risk of bias.

Nevertheless, despite reasonably consistent odds estimates in analyzed studies, the high I^2^ value (meta-analysis) suggests a significant heterogenicity. Applying the subgroups’ sensitivity verification, the exclusion of cross-sectional and case-control studies slightly attenuated PDS-ACI association due to deceased OR value. A single, moderate quality case-control study (low level of evidence) carried out by Gil-Montoya [147] markedly enhanced meta-analysis results as it found a strong association between periodontitis and ACI, reflected by a highest OR. However, the insufficient number of participants and short study duration was likely to influence the results.

### 4.1. Non-Clinical Data Supporting PDS-ACI Relationship

In the light of initial conclusion that a causal relation is not supported by clinical evidence, it can be reaffirmed that biological plausibility may derive from in vitro studies investigating various pro-inflammatory and neural parameters, including animal models [82,93,117,119]. Contrarily, some experts stated that “there may be some kind of association between periodontal pathogen *Porphyromonas gingivalis* and Alzheimer’s disease; causation is as yet a stretch too far” [156]. The proposed plausible interactions involve shared genetic risk determinants for PDS and ACI [157]. Although the results of some in vitro and animal model studies concluded that periodontitis could be an epidemiologically and biologically plausible risk determinant for cognitive deterioration/neurogeneration [158], the level of scientific evidence remains low. To date, it is not clear whether periodontitis, with its microbiological and inflammatory impact, can contribute to systemic pathogenesis. Nevertheless, non-clinical research projects are expected to justify further studies on PDS-ACI interconnection.

As experimental studies provide the strongest evidence of a causal relationship, they would be much desired in its evaluation applying the Bradford-Hill postulates [159]. Whilst interventional studies give the strongest evidence of a causal relationship, due to ethical reasons they must be robustly justified to be utilized for assessment of the relationship between periodontal status and neural system. Hence, randomized control trials, including periodontal treatment interventions and ACI or its markers as an outcome, could provide experimental evidence. Seven of the thirteen cohort studies were conducted in Asia, particularly in mixed Asian communities (Taiwan, South Korea, Japan) with the use of the same methods, i.e., national databases designed for national health insurance systems. Data collection bias and lack of data unification could impact the obtained results. Robust observational longitudinal clinical studies are needed, with particular attention paid to measurement of common confounders and detailed multivariable adjustment. These studies should include well-defined diagnostic criteria.

### 4.2. Causal Relationship Assessment Using Koch’s Postulates and Bradford-Hill’s Criteria

The relationship between the oral microbiome and cognitive impairment can be assessed for causality using Hill’s nine epidemiological considerations [35,136,159]. With limitations, the cumulative data only partially support a biological effect of periopathogens on ACI, particularly spirochetes versus Alzheimer’s disease [35,136] (Table S1). However, this finding does not preclude the causal association as per modern assessment using multi-level scoring system for causation. Only four of Bradford-Hill’s postulates are fulfilled after adjustment. The mechanisms of hypothetical link between periodontitis and ACI fall within the category of coherence and plausibility. One cohort study by Laugish et al. [160], assessing plasma biomarkers: tau protein, amyloid β, and antibodies level against selective periopathogens, does not support the hypothesis. Considering Bradford-Hill’s criteria, the cumulative data do not confirm a causal association between specific pathogens and Alzheimer’s disease and this claim is unsupported. The speculative nature of some studies exploring a hypothetical link between periodontitis and ACI was implicitly acknowledged by the authors.

### 4.3. Consistency of Temporality of Collected Data

Overall, the results of nine cohort studies moderately meet Bradford-Hill’s ‘consistency’ and ‘temporality’ criteria, plausibly indicating that a prolonged exposure to the primary determinant—periodontitis—seems necessary to observe a distinct association between PDS and ACI. Since the relevant studies demonstrated a long-term follow-up, more than 10 years, with the exposure to periodontitis preceding the ACI outcome, it can be stated that temporal relationship was ensured. Such a duration in follow-up would be needed in future trials. The temporal association seen in meta-analysis could justify the pivotal trial research. While the cognition function criteria were generally specified and uniform, the results suggest that well-defined criteria for periodontitis diagnosis are required to robustly determine a link between periodontitis and ACI. A similar concern was also raised by other authors [35,68].

### 4.4. Other Systematic Reviews Investigating the PDS-ACI Relationship

Previous SRs of potential link between periodontitis and ACI revealed deficiencies related to omission of suitable publications [124,161], inclusion of non-eligible studies [68], insufficient bias assessment [34,121] or lack of meta-analysis [121,162]. Nevertheless, findings from this analysis were mirrored in other pooled reviews in the same field. In multifaced systematic review combining 18 clinical and 10 in vitro and animal-based studies, Kamer et al. [35] concluded that interconnection between periodontitis and ACI fulfils the Bradford-Hill criteria for plausibility, analogy, and coherence. The observational studies provided evidence that partially supported the strength of association, consistency, and temporality criteria, although in selected cases, the periodontal disease exposure was not of primary interest [143]. It should be noted that SRs are not optimal unless all the confounders are identified and adequately controlled. Although Nadim et al. [68] claimed that moderate association between PDS and ACI exists (overall RR = 1.38, 95% CI 1.01–1.90; cohort studies RR = 1.18, 95% CI 1.06–1.31, and case-control studies RR = 2.25, 95% CI: 1.48–3.42), based on presented data, the level of evidence remains unverified.

Accordingly, the results of this study are in line with the most recent comparative subgroup analysis of twenty observational studies, carried out by Guo et al. [7], who found a strong relationship between periodontitis and cognitive decline (OR = 1.77, 95% CI: 1.31–2.38). The analysis of subgroups (severity of PDS) showed that moderate/severe periodontitis was associated with dementia (OR = 2.13). However, these reviews fail to account adequately for confounding and were characterized by different inclusion criteria, various design and arbitrary quality assessment tools, and heterogeneity of their integral components/protocols. The insufficient data synthesis found was one of the deficits affecting summarizing results in other systematic reviews. This observation is in line with the most recent SR [163], which found a very low level of evidence, suggesting a cautious interpretation of the results describing an association between periodontitis and neurodegenerative diseases.

### 4.5. Strengths and Limitations

This multifaceted review applied in-depth strategies to evaluate the quality of scientific evidence, with special attention paid to risk of bias. For a detailed comparison of potential confounders, the strict quantification of the data revealed how the quality of the primary data prevent attribution of a causal relationship, even though an association is evident. Herewith, the use of adjusted estimates from selected clinical studies in meta-analysis enhanced a robustness of systematic review. Fundamentally, the limitations of this review largely relate to the quality of the original studies. These deficiencies include inconsistent criteria applied for outcome and exposure, inadequately controlled confounding factors, subjects not representing the entire population, bias in control selection, adequacy of follow-up of cohort, and discrepancies in outcome measures. Some studies failed to demonstrate that the outcome of the interest was not present at the onset of study. Only three studies met representativeness criteria [132,133,137] to ensure inferential value. A lack of sample size estimation was observed in all studies. Most importantly, confounders adjustment was weak and inconsistent. Only two main confounders, age and sex, were adjusted accordingly, revealing a vast discrepancy regarding remaining socio-economic factors. Specifically, the residual effect of not correctly adjusted smoking and alcohol confounders would increase the risk of overestimation. Consequently, ACI may be a manifestation of other confounding factors responsible for compromised systemic responses that drive ACI, apart from periodontitis. The existence of complex confounding variables makes it difficult to establish a causal ACI-PDS relationship. Thus, it might be impossible to identify all confounders and remove them from the observed marginal associations to ascertain this relationship.

The potential impact of the severity of periodontitis on the magnitude of association could not be determined. First, variation in the classification of periodontitis has a potential impact on pooled data. This problem would be minimized by standardization of PDS recognition and diagnostics. The radiological parameter of periodontitis, alveolar bone loss as a main diagnostic measure, was used in only selected studies. The ICD-9-CM and ICD-10-CM classification and parameters used alone in five studies to diagnose PDS, different case definitions, and lacking assessment of essential periodontal parameters (pocket depth, alveolar bone loss), may not provide reliable clinical validity. Secondly, the coding parameters of ICD could affect the reproducibility of diagnosis. None of the studies under consideration used a new classification of PDS recommended by the European Federation of Periodontology (2017) [164].

### 4.6. The Implication of the Obtained Findings on Public Health Strategies

In the absence of evidence of a causal relationship between periodontitis and ACI, population-wide prophylaxis and treatment of PDS may not be pursued for the reduction of ACI. Controversially, Nadim et al. [68] suggested that a 50% reduction in the prevalence of periodontal disease may save, on average, 850,000 cases of dementia across the world, based on extrapolation of the cohort studies’ results. These claims are not evidence-based. As there are no sufficient evidence data, it is yet to be established if the treatment of periodontitis might be a part of prevention regime in persons belonging to high risk of systemic diseases. Adequately, treatment of periodontitis does not seem to be currently advocated in the prevention or management of ACI. If evidence supports population-based strategies such as improving oral health to minimize the likelihood of systemic disturbances in later life, health economic data would be required to demonstrate that treatment offered more benefits than the resources being used in another way, depending on emerging evidence. Public health programs have been already introduced to manage diabetes, in which PDS is deemed to play a role as a modifiable risk factor (European Federation of Periodontology, Madrid, Spain, 2017).

## 5. Conclusions

Considering the limitations of systematic review, the conclusion can be drawn that there is no direct evidence of a causal link between periodontitis and age-related cognitive impairment. The clinical evidence is not strong enough to support causality. Rendering shared risk factors and data synthesis results, the hypothesis that periodontitis and ACI are linked cannot be ruled out and a bidirectional relationship is possible. Weak evidence exists of a positive association between periodontitis and a higher risk of dementia or Alzheimer’s disease incidence. The magnitude of such evidence is inconsistent due to insufficient data which do not support the hypothesis that reduction of periodontitis prevalence and/or severity influences cognition outcome. Considering the global burden of cognitive decline disorders because of demographic changes, the screening of potential modifiable risk factors is predicted to be intensified as an integral part of multidisciplinary research efforts.

Whilst ACI is a major contributor to mortality, morbidity, and economic constraint in healthcare, the recognition of risk factors for its onset and progression should be prioritized. The associations observed in this study provide the impetus for high quality and appropriately designed, large-cohort clinical studies. Structured longitudinal trials, prompting control of various confounders, may bring new data, investigating whether periodontitis can be a potentially modifiable risk factor of ACI. The reduction of periodontal disease prevalence should be a target of global programs promoting oral health, regardless of its causal impact on systemic conditions.

## Figures and Tables

**Table 1:** Primary characteristics of 17 included studies.

Authors Year	Country, Settings	Sample Size (N) Gender	Age	Follow-Up (Years)	Primary Outcome
***Controlled Cohort studies***					
Lee, 2020 [132]	Taiwan, National Health Insurance Database	56,018 with newly diagnosed periodontitis, 56,018 without periodontitis	>50	13	Incidence of dementia.
Choi, 2019 [137]	South Korea, Korean National Health Insurance Service—Health database	Total 262,349; 46,344 with chronic periodontitis, 216,005 without periodontitis	>50; 60.2 mean age chronic periodontitis, 60.4 mean age healthy	10	Incident of Alzheimer’s disease and vascular dementia.
Iwasaki, 2019 [138]	Japan, Tosa Longitudinal Aging Study, Community-dwelling individuals	Total 179	>75; average 80.1	5	Diagnosis of mild cognitive impairment.
Nilsson, 2018 [145]	Sweden, Swedish National Study on Aging and Care	Total 715 (704 available for examination)	>60	6	Cognitive function deterioration
Chen, 2017 [133]	Taiwan, National Health Insurance Research Database	Total 18,672; 9291 with chronic periodontitis, 18,672 without chronic periodontitis (1:2 ratio)	>50; mean age 54.1 exposed cohort, mean age 54.2 unexposed cohort	10	Incidence of Alzheimer’s disease.
Lee, 2017 [134]	Taiwan, National Health Insurance Research Database	Total 6056; 3028 with periodontitis 3028 without periodontitis (age- and sex-matched)	>65; median age 72.42	10	New-onset dementia
Lee, 2017 [135]	Taiwan, National Health Insurance Research Database	182,747; 6133 with dementia	>45	10	Risk of newly developed dementia in individuals undergoing periodontal treatment
Tzeng, 2016 [136]	Taiwan, National Health Insurance Database	Total 8828 2207 with newly diagnosed periodontitis 6621 without periodontitis Sex and index year-matched control	>20	10	Risk of developing dementia
Iwasaki, 2016 [139]	Japan, Community-dwelling individuals	85; 21 with severe periodontitis 64 without severe periodontitis	>75; average 79.3, 79.5 with severe periodontitis, 79.3 without severe periodontitis	3	Cognitive function decline
Naorungroj, 2015 [141]	USA, Atherosclerosis Risk in Communities study	Total 911	52–75	8	Cognitive function change
Okamoto2015 [140]	Japan, Fujiwara-kyo study, Nara Prefecture	Total 2335; 241 developed mild memory impairment	>65; median age 71.0	5	Development of mild memory impairment
Stewart, 2013 [142]	USA, Health, Aging and Body Composition Random sample from Medicare databases	Total 3075; 1053 sample with full dental examination and cognitive assessment at Years 1 and 3, 2022 remainder of the baseline sample	70–79; mean 73.5–73.7	5	A primary outcome: global cognitive function decline
Arrive, 2012 [144]	France, Community based of elderly population	405; 72 developed dementia during the follow-up, 333 diagnosed with no dementia at the end of the follow-up	66–80; median age at baseline 70	15	The occurrence of dementia.
Kaye, 2010 [143]	USA, Veterans Affairs Dental Longitudinal Study	597 Men	28–84; median age: 45.5 at baseline	32 Dental examination conducted every 3 years	The cognitive function loss
***Cross-sectional***					
Holmer, 2018 [131]	Sweden, Huddinge muncipality	154 patients with Alzheimer’s disease (52), mild cognitive impairment (51), and subjective cognitive decline (51) 76 controls	50–80; median age 70 year—cases, medical age 67 years—controls	3	The risk of mild cognitive impairment, Alzheimer’s disease, and subjective cognitive decline
***Case-control***					
Stein, 2007 [146]	USA, Nun Study (Milwaukee)	Total 144; females, 76 cases with available radiographs to assess bone loss	75–98 years; Mean 84 years	10 Ten annual cognitive assessments	Cognitive function loss, neuropathological changes, and apolipoprotein E allele genotyping
Gil-Montoya, 2015 [147]	Spain, Granada, Neurology Departments, two hospitals	Total 409; 180 with cognitive impairment, 229 without cognitive impairment	51–98; mean age 77.00 cases, mean age 78.5 controls	2	The risk of cognitive impairment/dementia in newly diagnosed persons with and without periodontitis

**Table 2:** Main results of 17 studies investigating an association between PDS and ACI.

Authors Year	Aim(s) of the Study	Main Results and Conclusions
**Cohort studies**		
Lee, 2020 [132]	*“To compare the long-term risk of dementia in persons without and with periodontitis and its related factors”*	*“Higher incidence of dementia in patients with periodontitis, than without* (*5.19* vs. *3.02 per 1000 person/years*) *in men and females, especially among persons aged above 80 years (HR: 4.30)* *Estimated hazard ratio* (HR) = *1.73 (95% CI: 1.61–1.86)”*.
Choi, 2019 [137]	*“To assess the effect of chronic periodontitis on Alzheimer’s disease and vascular dementia occurrence”*	*“Patients with periodontitis had an increased risk of dementia and Alzheimer’s disease incident (adjusted HR 1.06 vs. 1.05, respectively). Chronic periodontitis associated with dementia after considering smoking, alcohol intake, and physical activity”.*
Iwasaki, 2019 [138]	*“To explore the association between a mild cognitive impairment and periodontitis and periodontal inflammation in older adults”*	*“Severe periodontitis was associated with mild cognitive impairment after adjusting for follow-up period, age, gender, smoking, education, physical activity, obesity, depression, and diabetes (adjusted OR 2.61; 95% CI: 1.08–6.28)”.*
Nilsson, 2018 [145]	*“To determine whether a diagnosis of periodontitis in individuals >60 years of age was associated with cognitive decline”*	*“A history of periodontal disease was an independent risk indicator for cognitive decline. Bone loss >4 mm at >30% of sites associated with cognitive decline after adjustment of main confounding factors. The unadjusted OR 2.8, 95% CI: 1.7–4.5, fully adjusted OR 2.2, 95% CI: 1.2–3.8 for age, education, and BMI”.*
Chen, 2017 [133]	*“To determine whether patients with chronic periodontitis are at risk of developing Alzheimer’s disease”*	*“Patients with periodontitis exposure exhibit a higher risk of Alzheimer’s disease occurrence (1.707-fold increase in the risk of developing Alzheimer’s disease, 95% CI: 1.152–2.528, adjusted HR)”.*
Lee, 2017 [134]	*“To determine whether periodontitis is a modifiable risk factor for dementia”*	*“Risk of dementia was higher in individuals with periodontitis (HR 1.16; 95% CI: 1.01–1.32) after adjustment for sociodemographic factors”.*
Lee, 2017 [135]	*“To determine the magnitude and temporal aspects of the effect of the poor oral health and periodontal disease on dementia”*	*“The incidence of dementia was significantly higher in persons with periodontal disease who did not receive treatment (0.76%) and who had teeth extracted due to periodontal disease (0.57%), compared to group that received intensive periodontal treatment (0.35%) and prophylaxis (0.39%) (HR 1.14, 95%, CI:1.04–1.24). Higher risk of dementia in persons with periodontitis who did not undergo periodontal treatmen”.*
Tzeng, 2016 [136]	*“To identify the association between chronic periodontitis and gingivitis, and the risk of developing dementia”*	*“The HR for dementia was 2.54 (95% CI: 1.297–3.352) after adjusting for main confounders: gender, age, income, urbanisation level, geographical region, and comorbiditites”.*
Iwasaki, 2016 [139]	*“To investigate whether periodontitis in elderly Japanese people could be a risk factor for cognitive decline”*	*“Severe periodontitis was significantly associated with risk of cognitive function decline (adjusted RR 2.2; 95% CI: 1.1–4.5). A 1.8-point greater decrease in MMSE score was observed in severe periodontitis (95%, CI: 3.3–0.2)”.*
Naorungroj, 2015 [141]	*“To determine whether tooth loss and current inflammatory state of periodontal disease predicted 8-year changes in cognitive function”*	*“Periodontal disease did not predict cognitive decline and was not associated with cognitive performance”.*
Okamoto, 2015 [140]	*“To investigate the effect of tooth loss on the development of mild memory impairment among elderly”*	*“No association between CPI code 0–3, code 4, and mild memory impairment”.*
Stewart, 2013 [142]	*“To investigate the relationship between periodontal disease and cognitive decline”.*	*“Worse periodontal status associated with cognitive impairment based on MMSE. Strong association between PPD/alveolar bone loss and cognitive impairment after adjustment for age and gender (OR 1.50 PPD vs 1.34 alveolar bone loss)”.*
Arrive, 2012 [144]	*“To examine the relationship between oral condition and occurrence of dementia in French older community dwellers”*	*“Periodontal condition was not associated with the risk of dementia”.*
Kaye, 2010 [143]	*“To determine whether tooth loss, periodontal disease, and caries incidence predict cognitive decline in men”*	*“The risk of low MMSE increased from 2% to 5% for each tooth with progression of alveolar bone loss or probing pocket depth”.*
**Cross-sectional studies**		
Holmer, 2018 [131]	*“To investigate the putative association among marginal periodontitis, cognitive impairment, and Alzheimer’s disease”.*	*“Alzheimer’s disease, mild cognitive impairment, and subjective cognitive decline were associated with more alveolar bone loss (OR 5.81; 95% CI: 1.14–29.68) and deep periodontal pockets (OR 8.43; 95% CI: 4.00–17.76). The strongest association was found between Alzheimer’s disease and the presence of one or more deep pockets > 6 mm (OR 15.12; 95% CI: 5.93–38.58)”.*
**Case-control studies**		
Gil-Montoya, 2015 [147]	*“To determine whether periodontitis is associated with the diagnosis of cognitive impairment/dementia after controlling for risk factors: age, gender, and education level”.*	*“Periodontitis indicators such as PPD and alveolar bone loss were significantly associated with diagnosis of cognitive impairment. The risk of cognitive impairment was above 3-fold higher in persons with severe periodontitis versus group with no or mild periodontitis after controlling for age, gender, and education”.*
Stein, 2007 [146]	*“To investigate the potential relationship between tooth loss and development of dementia”*	*“Alveolar bone loss was not associated with dementia at first cognitive examination (OR 1.5, 95% CI: 0.37–5.8) or incidence of dementia (HR 2.4, 95% CI: 0.86–6.6) after adjusting for age, education, and APOE4”.*

**Table 3:** Diagnostic criteria for periodontitis and cognitive impairment in 17 included studies.

Authors Year	Periodontitis Diagnosis; Criteria	Cognitive Impairment Diagnosis; Criteria
***Cohort studies***		
Lee, 2020 [132]	International Classification of Diseases, 9th revision, Clinical Modification (ICD-9-CM): ICD-9-CM 523.4,	International Classification of Diseases, 9th revision, Clinical Modification (ICD-9-CM): dementia code 290 and 331.0
Choi, 2019 [137]	International Classification of Diseases, 10th Revision (ICD-10): K05.3. At least one chronic periodontitis-related treatment such as: subgingival curettage, periodontal flap operation, gingivectomy, odontectomy	International Classification of Diseases, 10th Revision (ICD-10): codes F00, G30 for Alzheimer’s Disease or ICD-10 code F01 for vascular dementia
Iwasaki, 2019 [138]	Case definitions of periodontitis according to European Workshop in Periodontology Group C (EWP definition) and Centers for Disease Control/American Academy of Periodontology (CDC/AAP definition):	MMSE range from 0 to 30. Dementia is diagnosed according to the criteria of the DSM-IV. Patients diagnosed with mild cognitive impairment if they fulfilled criteria: (1) subjective cognitive complaints, (2) memory problems that were abnormal for the patient’s age, (3) preserved functional activity of daily living, (4) failure to meet the DSM-IV criteria for dementia
Nilsson, 2018 [145]	PPD at four sited of existing teeth > 5 mm on 30% of teeth Alveolar bone loss >4 mm from CAJ to marginal bone level on >30% of readable sites.	Mini-Mental Stage Examination (MMSE) as cognitive outcome variable
Chen, 2017 [133]	ICD-9-CM code 523.4 (chronic periodontitis).	ICD-9-CM code 331.0, Alzheimer’s disease, dementia
Tzeng, 2016 [134]	ICD-9-CM, codes 523.1 (chronic gingivitis), and 523.4. (chronic periodontitis).	ICD-9-CM, codes for dementia: 290.20–290.10–290.13, 290.20–290.21, 290.3, 331.0, 290.41–290.43, 290.8–290.9. Alzheimer-type dementia: ICD-9-CM 290.0, 290.10–290.13, 290.20–290.21, 290.3, 331.0. Vascular dementia: ICD-9-CM 290.41–290.43. Non-vascular dementia: ICD-9-CM 290.8–290.9 Diagnostic and Statistical Manual of Mental Disorders, 4th Edition Revised (DSM-IV)
Iwasaki, 2016 [135]	Case definitions of periodontitis according to Centers for Disease Control/American Academy of Periodontology (CDC/AAP definition): - severe periodontitis: >2 interproximal sites with AL of >6 mm (not on the same tooth) and >1 interproximal site with PPD > 5 mm.	MMSE as cognitive functioning test by trained psychologists Cognitive decline if <24 score
Lee, 2017 [136]	ICD-9-CM, codes 523.3-5	ICD-9-CM, codes 290.0-290.4, 294.1, 331.0-331.2
Lee, 2017 [139]	ICD-9-CM code 523.0–523.5, subjects diagnosed with gingivitis, gingival recession, acute, or chronic periodontitis. Four groups: Group 1—only dental prophylaxis (scaling) required, Group 2—intensive periodontal treatment (subgingival curettage, root planning, periodontal flap operation) required. Group 3—tooth extraction required due to periodontal disease, Group 4—subjects who did not receive any of these treatments	ICD-9-CM code 290.X, 331.0. Subjects with diagnosis of presenile dementia, vascular dementia, senile dementia, Alzheimer’s disease.
Naorungroj, 2015 [141]	PPD, BOP BGI based on PPD and BOP	Delayed word test (DWT), Digital symbol substitution (DSS), Word fluency (WF)
Okamoto, 2015 [140]	CPI (baseline code 0–3 and code 4)	MMSE score assessed by a specialist psychiatrist or clinical psychologists
Stewart, 2013 [142]	PPD (mean value and proportion with >3 mm depth) AL (>3 mm; defined as the mean number of sites affected and the proportion of sites examined that were affected)	Modified Mini-Mental State Examination (3MSE) used for an assessment of global function. Digit Symbol Substitution test measuring attention, psychomotor speed, and executive function. Clock drawing test used to measure executive function
Arrive, 2012 [144]	CPI (cut-off code 3 and 4)	Neuropsychological testing: DSM-III R for dementia. National Institute of Neurological and Communication Disorders and Stroke/Alzheimer’s Disease and Related Disorders Association (Alzheimer’s disease criteria) Hachinski score (vascular dementia criteria)
Kaye, 2010 [143]	PPD, Alveolar bone loss Number of teeth with PPD > 4 mm Number of teeth with Alveolar bone loss	MMSE: low cognitive status, mild cognitive impairment < 25 points of the age or education—specific median on the MMSE Spatial Coping Task—Developmental Test of Visual-Motor Integration
***Cross-sectional studies***		
Holmer, 2018 [131]	PPD Number of teeth with PPD 4–5 mm and with PPD > 6 mm Alveolar bone loss based on OPT: no or mild, localised, generalised	ICD, 10th Revision. MMSE > 20 for patients diagnosed with Alzheimer’s disease. Winblad criteria for patients diagnosed with mild cognitive impairment. Pre-Mental Cognitive Impairment criteria for patients diagnosed with subjective cognitive decline
***Case-control studies***		
Gil-Montoya, 2015 [147]	PPD, AL Degree of periodontitis—percentage of sites with AL > 3 mm	Behavioural assessment (neuropsychiatric inventory scale) Clinical dementia rating scale. DSM—IV for dementia from National Institute of Neurological and Communicative Disorders and Stroke-Alzheimer’s Disease and Related Disorders Association
Stein, 2007 [146]	Alveolar bone loss based on OPT: moderate and severe bone loss due to low number of subjects with severe periodontitis	Cognitive function assessed annually by MMSE and Activities of Daily Living, Delayed Word Recall Test, Boston Naming Test, verbal fluency test, construction praxis test. Criteria considered to be diagnosed with dementia: impairment in memory, impairment in at least one other area of cognition, impairment in social or daily function

PD—Periodontal Pocket Depth, AL—Attachment Loss, BOP—Bleeding on Probing, ABL—Alveolar Bone Loss, OPT—Orthopantomogram, CPI—Community Periodontal Index.

**Table 4:** Quality of cohort studies assessed using the Newcastle–Ottawa scale.

Study	Selection of Cohort				Comparability of Cohorts		Outcome			
	Representativeness of the Exposed Cohort	Selection of the Non-Exposed Cohort	Ascertainment of Exposure	Demonstration that Outcome of the Interest Was Not Present at Start of Study	Age and Gender/ Additional Factor		Assessment of Outcome	Long Enough Follow-Up for Outcome to Occur	Adequacy of Follow-Up of Cohorts	Total
Lee, 2020 [132]	*	*	*		*		*	*		6/9
Choi, 2019 [137]	*	*	*		*	*	*	*		7/9
Iwasaki, 2019 [138]		*	*	*	*	*	*	*		7/9
Chen, 2017 [133]	*	*	*		*		*	*		6/9
Lee, 2017 [134]		*	*		*		*	*		5/9
Tzeng, 2016 [136]		*	*		*		*	*		5/9
Iwasaki, 2016 [139]		*	*	*	*	*				5/9
Stewart, 2013 [142]		*	*	*	*	*				5/9
Naorungroj, 2015 [141]		*	*	*	*	*		*		6/9
Okamoto, 2015 [140]		*	*	*	*	*	*			6/9
Arrive, 2012 [144]			*		*	*		*	*	5/9
Kaye, 2010 [143]			*		*	*	*	*	*	6/9

**Table 5:** Quality of cross-sectional studies assessed using the Newcastle–Ottawa scale.

Study	Selection				Comparability		Outcome		
	Representativeness of the Sample	Sample Size	Non-Respondents	Ascertainment of the Exposure (Absence or Exclusion)	The Subjects in Different Outcome Groups Are Comparable, Based on the Study Design or Analysis. Confounding Factors are Controlled.		Assessment of Outcome	Statistical Test	Total
Lee, 2017 [134]		*		*	*	*	*	*	6/8
Holmer, 2018 [131]				*	*	*	*	*	5/8

**Table 6:** Quality of case-control studies assessed using the Newcastle–Ottawa scale.

Study	Selection of Case-Control				Comparability		Exposure			
	Is the Case Definition Adequate?	Representativeness of the Cases	Selection of Control	Definition of Control	Comparability of Cases and Controls on the Basis of the Design or Analysis		Ascertainment of Exposure	Same Method of Ascertainment for Cases and Controls	Non-Response Rate	Total
Gill-Montoya, 2015 [147]	*			*	*		*	*		5/9
Stein, 2007 [146]			*		*	*	*	*		5/9

**Table 7:** Methods used to account for confounders in 17 included studies.

	Confounders Accounted for	Methods to Account
**Cohort studies**		
Lee, 2020 [132]	Age, gender, income, coexisting medical conditions, period of follow-up	Stratification
Choi, 2019 [137]	Smoking, physical activity, alcohol consumption	Stratification
Iwasaki, 2019 [138]	Age, gender, smoking status, educational level, physical activity, obesity, depression, diabetes	Multilevel mixed-effects logistic regression
Nilsson, 2018 [145]	Age, gender, level of education, use of interdental aids, frequency of dental visits, living alone, diabetes, ischaemic heart disease, BMI, dental caries	Logistic regression
Chen, 2017 [133]	Age, gender, comorbidities, Charlson comorbidity index, and urbanisation level. Patients were matched according to gender, age, and index years	Cox regression models adjusted for Alzheimer’s disease-related comorbidities, CCI, and urbanisation level
Lee, 2017 [134]	Adjustment for age, gender, for socio-demographic characteristics and comorbidities	Age and gender-matched control groups
Lee, 2017 [135]	Age, gender, socio-economic status, comorbidities	Cox regression model
Tzeng, 2016 [136]	Adjustment for age, gender, income, urbanisation level, geographic region, comorbidities	Age and gender-matched control Multivariate Cox proportional hazards regression analysis
Iwasaki, 2016 [139]	Age, gender, smoking, alcohol, education, depression, BMI, exercise, comorbidities	Multivariable regression
Naorungroj, 2015 [141]	Age, gender, smoking, alcohol, education, comorbidities, BMI, APOE4 allele genotype	Logistic regression, bivariate analysis
Okamoto, 2015 [140]	Age, gender, smoking, alcohol, education, comorbidities	Multivariable logistic regression
Stewart, 2013 [142]	Adjustment for age, gender, education, race, depressive symptoms, cardiovascular risk. Separate exploratory adjustments for inflammatory markers, BMI, anticholinergic medications use	Initial logistic regression models, followed by further adjustments. Stratification to investigate effect modification according to apolipoprotein E genotype
Arrive, 2012 [144]	Age, gender, smoking, alcohol intake, vascular risk factors, depressive symptoms	Multivariate analyses
Kaye, 2010 [143]	Age, years of education, smoking, alcohol intake, BMI, comorbidities	Multivariable models Cox proportional hazards regression models stratified according to median age
**Cross-sectional studies**		
Holmer, 2018 [131]	Age, gender, education, smoking, marital status, BMI, diabetes	Age- and gender-matched controls Multivariable regression models Sensitivity analysis for unmeasured confounding
**Case-control studies**		
Stein, 2007 [146]	Age, gender, education	Multivariable logistic regression
Gil-Montoya, 2015 [147]	Age, gender, education level, oral hygiene habits adjusted for main potential confounders	Multiple logistic regression

**Table 8:** Potential, co-existing residual confounders in 17 included studies.

Study	Smoking	Alcohol	Education	Socio-Economic Status
**Cohort studies**				
Lee, 2020 [132]	-	-	-	Low income: yes/no
Choi, 2019 [137]	Never smoker Past smoker Current smoker	[Times per week]. None. 0–1, 1–2, 3–4, >5	-	Household income: first, second, third, fourth quartile
Iwasaki, 2019 [138]	Never smoker Previous smoker Current smoker	Not. Daily drinker	Less education, 6 years of school attendance	-
Nilsson, 2018 [145]	Never smoker Current, Former	Yes/No	Elementary Higher	-
Lee, 2017 [134]	-	-	-	-
Lee, 2017 [135]	-	-	-	<20,000, 20,000–39,999 >40,000
Chen, 2017 [133]	-	-	-	-
Tzeng, 2016 [136]	-	-	-	Monthly income
Iwasaki, 2016 [139]	Never Former smoker Current smoker	Yes/No	Lower education: school attendance for less than 7 years	-
Naurungroj, 2015 [141]	Cigarette use Current Former Never	Current Former Never	Less than high school High school completion Postsecondary education	Refused <25,000$ $25 < 50,000 $50,000 or more
Okamoto, 2015 [140]	Never Former smoker Current	Hardly drink Drink at least one day a week	Less than 12 years, more than 12 years	-
Stewart, 2013 [142]	Never Former smoker Current smoker	-	<High school. High school graduate. Postsecondary	-
Arrive, 2012 [144]	Tobacco consumption: yes/no	Alcohol intake: yes/no	Higher school level: yes/no	-
Kaye, 2010 [143]	Never Ever smoked	Alcohol intake [g/day]	Years of education	-
**Cross-sectional studies**				
Holmer, 2018 [131]	Never Previous smoker Current smoker	-	1–2 years University	Annual income
**Case-control studies**				
Stein, 2007 [146]	-	-	Similar level of education (nuns)	-
Gil-Montoya, 2015 [147]	Tobacco: yes/no, <20 and >20 cig./day	Alcohol intake: yes/no, 1–6 times a week, >1 times/day	High. Primary. Primary incomplete	-

## Data Availability

The raw data spreadsheets are available on request.

## Supplementary Materials

The following supporting information can be downloaded at: https://www.mdpi.com/article/10.3390/ijms232315320/s1, References are cited in [165,166,167,168,169,170].
