# Supplementary material for: Is Periodontitis Associated with Age-Related Cognitive Impairment? The Systematic Review, Confounders Assessment and Meta-Analysis of Clinical Studies
Source: Int J Mol Sci. 2022 Dec 5;23(23):15320. doi: 10.3390/ijms232315320 (PMC9739281; doi:10.3390/ijms232315320)
Supplement: Supplementary file 1 [file ijms-23-15320-s001.zip › ijms-2036687-supplementary.pdf]

Table S1. Oral microorganisms potentially involved in aetiology of cognitive impairment

Year, authors	Method	Periodontal microorganisms	Findings
2002, Riviere et al. [119]	Experimental, postmortem, brain tissue, PCR, immunochemistry	*Treponema*	Detected
2009, Kammer [60]	Observational, blood, TNF-α, IL-1β, IL-6 levels, IgG antibody	*T. denticola, P. gingivalis, A. actinomycetemcom.*	Correlation between the detected level of antibodies and Alzheimer's disease diagnosis
2012, Sparks et al. [165]	Longitudinal, blood, antibodies level	*A. actinomycetemcom. P. gingivalis, C. rectus, T. denticola, Fusobacterium nucleatum, T. forsythia*	Relationship between antibodies level and onset/progression of Alzheimer's disease
2013, Poole et al. [70]	Experimental, postmortem, brain tissue, cell line SVGp12, bacterial lipopolisacharydes (LPS)	*T. denticola* *T. forsythia* *P. gingivalis* (P.g.)	Underpins the hypothesis of association between LPS produced by P.g. and Alzheimers' disease
2014, Noble et al. [75]	Case-cohort study, plazma level of IgG in persons with Alzheimer's disease	*P. gingivalis, T. forsythia A.actinomycetemcom. T. denticola, C. rectus, E. nodatum*	Plazma IgG level against periodontal bacteria is associated with risk for developing incident of Alzheimer's disease
2016, Ide et al. [41]	Cohort, observational study, plazma antibodies, inflammatory markers, DNA assay	*P. gingivalis*	Results indicate a relationship between periodontitis and cognitive impairment
2017, Wu et al. [133]	Experimental, animals, (mice), MG6 microglia cell line, LPS	*P. gingivalis*	Results support the infectious hypothesis of Alzheimer's disease
2017, Liu et al. [166]	Experimental, animals, (mice), MG6 microglia cell line, P.g. gingipain	*P.gingivalis*	Results support the infectious hypothesis of Alzheimer's disease
2017, Ishida et al. [167]	Experimental, animals (mice), amyloid β, IL-1β, TNF-α	*P. gingivalis*	Periodontitis is a risk factor of Alzheimer's disease
2019, Hayashi et al. [168]	Experimental, animal (mice), LPS	*P. gingivalis*	Exposure to LPS affects prognosis of Alzheimer's disease
2019, Nie et al. [169]	Experimental, animal (mice), IL-1β, amyloid β	*P. gingivalis*	Potential role in novel therapeutic agents inhibiting P.g. and the onset of Alzheimer's disease
2019, Diaz-Zuniga et al. [170]	Experimental, animal (rat, cell line), IL-1β, IL-6, TNF-α, amyloid β	*A.actinomycetemcom.*	Probable association between *Aggregatibacter* periopathogen and Alzheimer's disease etiology
2019, Dominy et al. [117]	Prospective pilot study, animal (mice) and patient with Azheimer's disease, P.g. gingipain, P.g. DNA, amyloid β	*P. gingivalis*	*P. gingivalis* and its exo-toxin gingipain play a role in pathogenesis of Alzheimer's disease

Table S2. Database search criteria

1#

(periodontal* OR periodontium OR periodontitis OR periodontal disease OR chronic periodontitis OR periodontal pocket OR probing depth OR pocket depth OR clinical attachment loss OR alveolar bone loss OR periodontal bone loss OR attachment loss OR tooth mobility OR alveolar resorption OR bone loss)

AND

2#

(cognitive* OR cognition OR cognitive function OR memory OR memory loss OR cognitive impairment OR cognitive decline OR Alzheimer disease OR Alzheimer's* OR dementia OR vascular dementia OR neurodegeneration OR neurodegenerative disease OR central nervous system OR brain OR neural OR neurological)

Table S3. Worksheet of extracted details from eligible articles.

	General information				Methods	Population										Exposure and Control				Cognitive impairment (CI)			
N	Authors Year	Title Reference	Country Region	Source of study sample: (community, database, hospital, university)	Study deisign Length of follow-up (years)	Sample size (N)			Age at baseline (years)		Gender N (%) or NA (if excluded)		Controlled main confounders (Yes/No)		Health status at baseline	Case definition selected for periodontitis. NA in case of absence	Assessment of periodontal status	Periodontal status at baseline N (%) or NA		Case definition selected for CI. NA in case of absence	Assessment of cognitive status	Cognitive status at baseline N (%) or NA	
						Baseline	Drop-outs	Analyzed	Range (min-max)	Mean	Men	Women	Age	Gender	1. General - both systemically healthy and diseased. 2. Only systemically healthy people.	Case definition selected for periodontitis. NA in case of absence	1. Clinical. 2. Radiological NA. Periodontitis not assessed	No Periodontitis	Periodontitis	Case definition selected for CI. NA in case of absence	1. Cognitive tests 2. Only diagnosis NA. Cognitive status not fully assessed	No CI	CI

Table S4. Characteristics of main confounders and covariates in 17 reviewed studies.

Study	Age	Sex	Smoking Alcohol	Comorbidities	Medications	BMI	Education	Income	Physical activity	Oral hygiene Dental care	Other
**Cohort studies**											
Lee, 2020 [132]	+	+	-	Hypertension, Mental disorders, Diabetes, Ischaemic heart disease, Stroke, COPD, Liver cirrhosis, Dialysis, Traumatic brain injury, Hyperlipidaemia	+	-	-	+	-	-/-	-
Choi, 2019 [137]	+	+	+S/A	Systolic blood pressure, Fasting serum glucose, Total cholesterol Charlson comorbidity index	-	+	-	+	+	-/-	-
Iwasaki 2019 [138]	+	+	+S/A	Hypertension, Depression, Diabetes, Hearing loss	-	+	+	-	+	-/-	-
Nilsson, 2018 [145]	+	+	+S	Ischaemic heart disease, Diabetes	-		+	-	-	+/+	Living alone
Chen, 2017 [133]	+	+	-	Hypertension, Stroke, Depression, Diabetes Hyperlipidaemia, kidney disease, Traumatic brain injury	-	-	-	-	-	-/-	Urbanisation level
Lee, 2017 [134]	+	+	-	Hypertension, Cardiovascular disease Stroke, Diabetes, Chronic kidney disease	-	-	-	-	-	-/-	Urbanisation level
Lee, 2017 [134]	+	+	-	Hypertension, Diabetes Hyperlipidemia	-	-	-	+	-	-/-	Residential urbanicity
Tzeng, 2016 [136]	+	+	-	Hypertension, Stroke, Hyperlipidaemia, Depression, Traumatic brain injury, Diabetes Chronic kidney disease	-	+	-	+	-	-/-	Insured premium

Iwasaki, 2016 [139]	+	+	+S/A	Hypertension, Stroke, Cardiovascular disease, Depression, Diabetes	-	+	+	-	+	-/-	-
Naorungroj, 2015 [141]	+	+	+S/A	Hypertension, Coronary heart disease Diabetes, Stroke, Hyperlipidemia	-	+	+	+	-	-/-	APOE4
Okamoto, 2015 [140]	+	+	+S/A	Hypertension, Cerebrovascular disease, Myocardial infarction Diabetes, Cancer, Dyslipidaemia	-	-	+	-	-	-/-	-
Stewart, 2013 [142]	+	+	+S	Coronal heart disease, Cerebrovascular disease, Hypertension, Diabetes	+	+	+	+	-	-/-	CRP, IL6, APOE4
Arrive, 2012 [144]	+	+	+S/A	Hypertension, Angina, myocardial infarction, Stroke, Diabetes, Depression	-	+	+	-	-	-/-	Living alone
Kaye et al., 2010 [143]	+	-	+S/A	Hypertension, Cardiovascular disease Stroke, Diabetes	+	+	+	-	-	-/-	-
**Cross-sectional studies**											
Holmer, 2018 [131]	+	+	+/S	Number of comorbidities	+	+	+	+	-	+/+	Marital status
**Case-control studies**											
Stein, 2007 [146]	+	+	-	-	-	-	+	-	-	-/-	-
Gil-Montoya, 2015 [147]	+	+	+S/A	Hyperlipidaemia Hyperglycaemia	-	-	+	-	-	+/-	-
